# Cold stress in the harvest period: effects on tobacco leaf quality and curing characteristics

**DOI:** 10.1186/s12870-021-02895-w

**Published:** 2021-03-08

**Authors:** Yan Li, Ke Ren, Mengyang Hu, Xian He, Kaiyuan Gu, Binbin Hu, Jiaen Su, Yan Jin, Wenyou Gao, Daosheng Yang, Folin Li, Congming Zou

**Affiliations:** 1 Yunnan Academy of Tobacco Agricultural Sciences, Kunming, Yunnan People’s Republic of China; 2 Dali Tobacco Monopoly Bureau of Yunnan Province, Dali, Yunnan People’s Republic of China; 3 College of Tobacco Science, Yunnan Agricultural University, Kunming, Yunnan People’s Republic of China

**Keywords:** Flue-cured tobacco, Abiotic stress, Nitrogen metabolites, Carbon metabolites, Physiology and biochemistry, Enzyme activity

## Abstract

**Background:**

Weather change in high-altitude areas subjects mature tobacco (*Nicotiana tabacum* L.) to cold stress, which damages tobacco leaf yield and quality. A brupt diurnal temperature differences (the daily temperature dropping more than 20 °C) along with rainfall in tobacco-growing areas at an altitude above 2450 m, caused cold stress to field-grown tobacco.

**Results:**

After the flue-cured tobacco suffered cold stress in the field, the surface color of tobacco leaves changed and obvious large browning areas were appeared, and the curing availability was extremely poor. Further research found the quality of fresh tobacco leaves, the content of key chemical components, and the production quality were greatly reduced by cold stress. We hypothesize that cold stress in high altitude environments destroyed the antioxidant enzyme system of mature flue-cured tobacco. Therefore, the quality of fresh tobacco leaves, the content of key chemical components, and the production quality were greatly reduced by cold stress.

**Conclusion:**

This study confirmed that cold stress in high-altitude tobacco areas was the main reason for the browning of tobacco leaves during the tobacco curing process. This adverse environment seriously damaged the quality of tobacco leaves, but can be mitigated by pay attention to the weather forecast and pick tobacco leaves in advance.

**Supplementary Information:**

The online version contains supplementary material available at 10.1186/s12870-021-02895-w.

## Background

Cold stress greatly influences crops growing in high-altitude areas, so it is essential to explore the mechanism of natural cold stress to field crops [1]. Tobacco (*Nicotiana tabacum* L.) is extremely sensitive to low temperature. The growth of tobacco plants is restricted at the temperature lower than 10 to 13 °C and plants will die when the temperature drops to 2 to 3 °C [2]. Therefore, tobacco is suitable to be studied as a model plant for crops subjected to cold stress. The most suitable temperature in the field growth phase of tobacco is between 22 and 28 °C and the temperature should not be lower than 20 °C in the mature period. High-quality tobacco leaf production is greatest between 22 and 25 °C. Yunnan Province in China is located on a low-latitude plateau under the influence of significant mountain and monsoon climates. Cold stress significantly influences Yunnan agriculture. Generally, August is the later stage for growth of flue-cured tobacco in Yunnan, but the occurrence of low temperature often covers the entire tobacco growing season, which seriously affects the quality of fresh leaves. The low temperatures that cause cold stress to plants are still above freezing, and the damage to crops is mainly reflected in cell dehydration, damage to the plasma membrane system, and disorder of enzyme activity, which in turn damages the leaf physiological and biochemical systems [3].

Crops subjected to cold stress change in appearance to present chlorosis and flavescence as well as wilting and leaf shrinkage [4]. Moreover, stunted growth and development can make tobacco plants short, small, thin, and weak; leaves are found to have necrotic spots; the germination rate and seed setting rate decrease; eventually, the plant populations develop in disequilibrium [5]. If cold stress occurs in critical periods (e.g. booting–grouting period) of rice, for example, the crop yield can drop by 25%. However, existing research on cold stress in tobacco (an important leaf-use economic crop) has rarely been reported. The mechanism underlying field cold stress on tobacco in the harvest period would be useful production knowledge.

Tobacco under cold stress shows one key feature: growth is inhibited. The feature is reflected in agronomic traits and the quality of fresh tobacco leaves. Cold stress could reduce the agronomic traits such as plant height, leaf number and stem circumference of flue-cured tobacco, thus affecting the morphological formation of flue-cured tobacco. The quality of fresh tobacco leaves is an importantly influences to curing characteristics and tobacco yield and quality [6]. Cold stress can change the quality of fresh tobacco leaves by affecting the microstructures, chloroplast pigment content and maturity of tobacco leaves, further reduced the curing characteristics of tobacco leaves. The curing characteristics include yellowing due to water loss and leaf-drying of tobacco leaves as well as mutual compatibility of various trends [7, 8]. Existing data reveals that tobacco leaves undergoing cold stress will show great imbalance between water loss and yellowing and the formation of large-areas scalded tobacco in the curing process [9].

The difference in the chemical composition of tobacco leaves reflects specific differences in carbon and nitrogen metabolism; temperature affects the carbon and nitrogen metabolism of flue-cured tobacco. Cold stress also greatly decreases the root activity of tobacco and inhibits soil nitrogen uptake, thus impairing carbon and nitrogen metabolism [10]. As the product of secondary plant metabolism, polyphenols are important substances regulating tobacco leaf quality. Tobacco seedlings contain more phenylalnine ammonialyase (PAL) and promote polyphenol accumulation in cold stress. The content of a downstream product, lignin, can significantly increase stress resistance [11, 12].

Tobacco has developed a set of defense and protection systems to resist stress. Physiological and biochemical indices not only reflect the damage to such defense and protection systems for tobacco but also mirror its capacity of resisting stress. Research indicates that the antioxidant enzyme system will be activated when tobacco undergoes cold stress. Under the synergistic effect, the active oxygen in tobacco is eliminated so it can maintain at a low level, thus reducing the damage to plants [13, 14]. In this case, the activities of malondialdehyde (MDA) and peroxidase (POD) are greatly enhanced while those of superoxide dismutase (SOD) and catalase (CAT) are significantly lowered.

Laojun Mountain Town is in the northwest of Jianchuan County, Dali Bai Autonomous Prefecture of Yunnan Province in a high-altitude intermountain basin, with an average altitude of 2450 m, an annual average air temperature of 10 °C, annual precipitation of 960 mm, sunshine duration of 2200 h, a frost duration of 210 to 220 d, a heavy frost period of about 90 d, and frequent hail in summer and autumn. Tobacco is one of the major economic crops and Honghuadajinyuan (HD) is the main variety, showing favorable cold hardiness. According to 2019 data, the planting area of tobacco in Laojun Mountain town has reached 173.2 ha^− 1^, with a yield of 1950 to 2250 kg ha^− 1^ and an average price of 4.93 dollar kg^− 1^. The area subjected to cold stress is about 64 ha^− 1^, which accounts for 37% of the total planting area. During cold stress, some middle leaves and the majority of the upper leaves of tobacco cannot be cured, which results in the direct economic loss of 184,000 dollars to local tobacco growers.

Existing reports concentrate on exploring the changes of appearance and physiological and biochemical indices of tobacco seedlings in the environment of artificial cold stress. Related research into cold stress of field-grown tobacco in the harvest period remains rare. We hypothesize that cold stress can destroy the antioxidant enzyme system of mature tobacco leaves reducing the quality of fresh tobacco leaves, the content of key chemical components, and the production quality by aggravating the occurrence of browning tobacco during tobacco curing. To test our hypothesis, we observed that cold damage occurred frequently in the mature period of flue-cured tobacco in Dali of Yunnan province, leading to serious economic losses to tobacco farmers. We analyzed the meteorological data of Yunnan province in five recent years and found that the temperature in Jianchuan County of Dali of Yunnan Province fluctuated irregularly from July to September each year, with frequent low temperature above zero (Fig. S1). We chose the unique natural climate conditions of Yunnan Province to investigate the mechanism underlying cold stress of field-grown tobacco by making side-by-side comparisons of field grown (stressed) and greenhouse-grown (unstressed) tobacco. We expect to provide an empirical basis for the inducing factors and mechanism underlying the cold stress on field-grown tobacco in the harvest period and suggest feasible methods to reduce the economic loss suffered by tobacco growers.

## Results

### Effects of meteorological factors on field cold stress of flue-cured tobacco

**Fig. 1:**
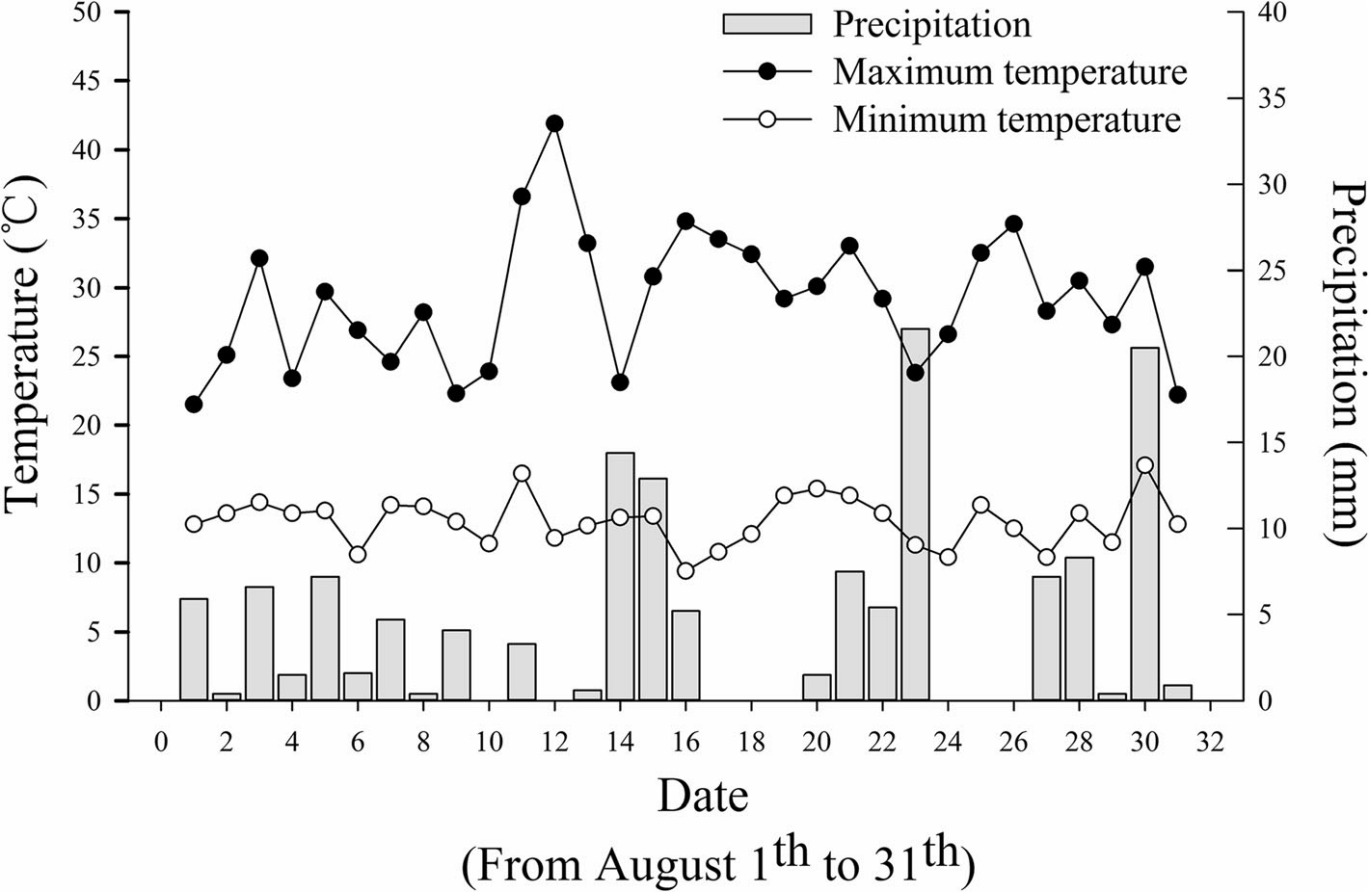
Daily precipitation and the daily temperature fluctuation from 1 to 31 August in Laojun Mountain Town, Jianchuan County

The temperature and rainfall fluctuated obviously in August at the experiments site (Fig. 1). On 12 August, the maximum diurnal temperature difference (30.1 °C) occurred but there was no rain. According to the results of local field cold stress investigation, there was a significant drop temperature accompanied rainfall and a little hail occurred on 16 and 17 August. In the next days, tobacco showed a range of symptoms caused by cold stress [15]. The surface color of the tobacco leaves changed from normal to dark green, purple red, then dark red, and finally off-white [15]. Finally, there was a large area of scalded leaves appeared, showing poor flue-curing availability and poor quality of flue-cured tobacco leaves.

**Fig. 2:**
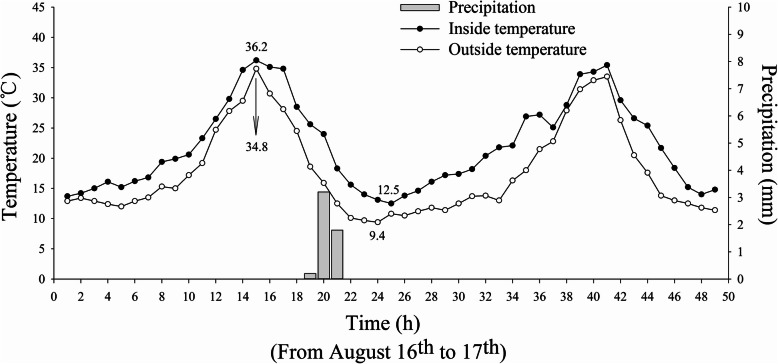
Temperatures inside and outside the greenhouse and precipitation during cold stress in Jianji Village, Laojun Mountain Town (16 to 17 August)

**Fig. 3:**
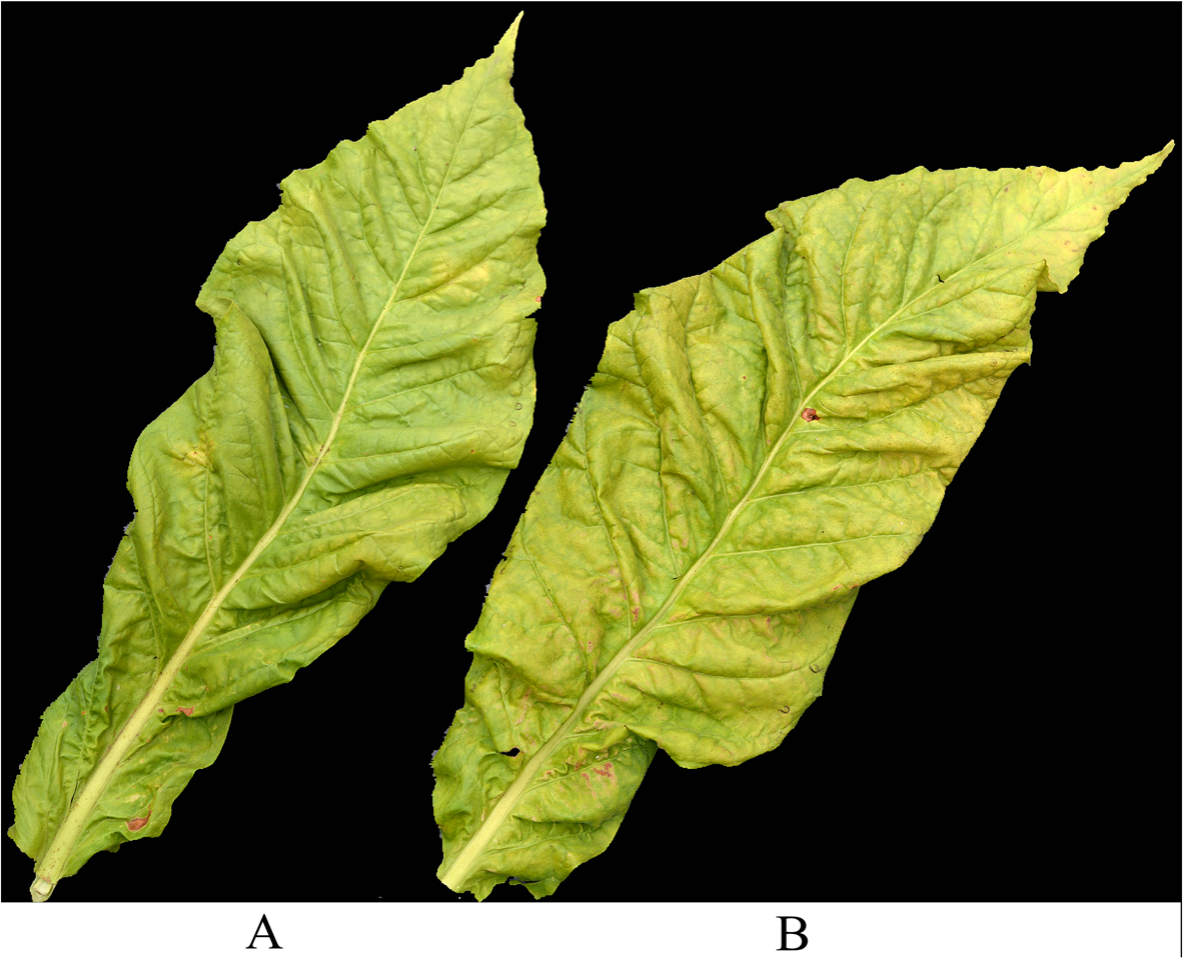
A comparison of sampled tobacco leaves inside and outside the greenhouse. **a** Fresh tobacco leaves inside the greenhouse on 19 August. **b** Fresh tobacco leaves outside the greenhouse on 19 August

To explore the specific causes for cold stress, the meteorological data collected inside and outside the greenhouse on 16 to 17 August were analyzed (Fig. 2). The temperature outside the greenhouse (natural conditions) varied from 9.4 to 34.8 °C. The temperature inside the greenhouse varied between 12.5 to 36.2 °C. Rainfall occurring in the cooling period was concentrated between 18:00 to 22:00 on 16 August, with a total precipitation of 5 mm. The tobacco leaves inside and outside the greenhouse were sampled on 19 August (Fig. 3). The 10th leaf (counting from bottom to top) was sampled. Tobacco leaves inside the greenhouse showed no significant symptoms of cold stress, except for a small number of brown spots on the leafstalk (Fig. 3); tobacco leaves outside the greenhouse exhibited significant red and puce plaques from the middle part of the leaves to the leafstalk and the leaves turned from green to yellow on the whole. It was judged that tobacco leaves outside the greenhouse were subjected to cold stress.

### Comparison of slices of tobacco leaves inside and outside the greenhouse

**Fig. 4:**
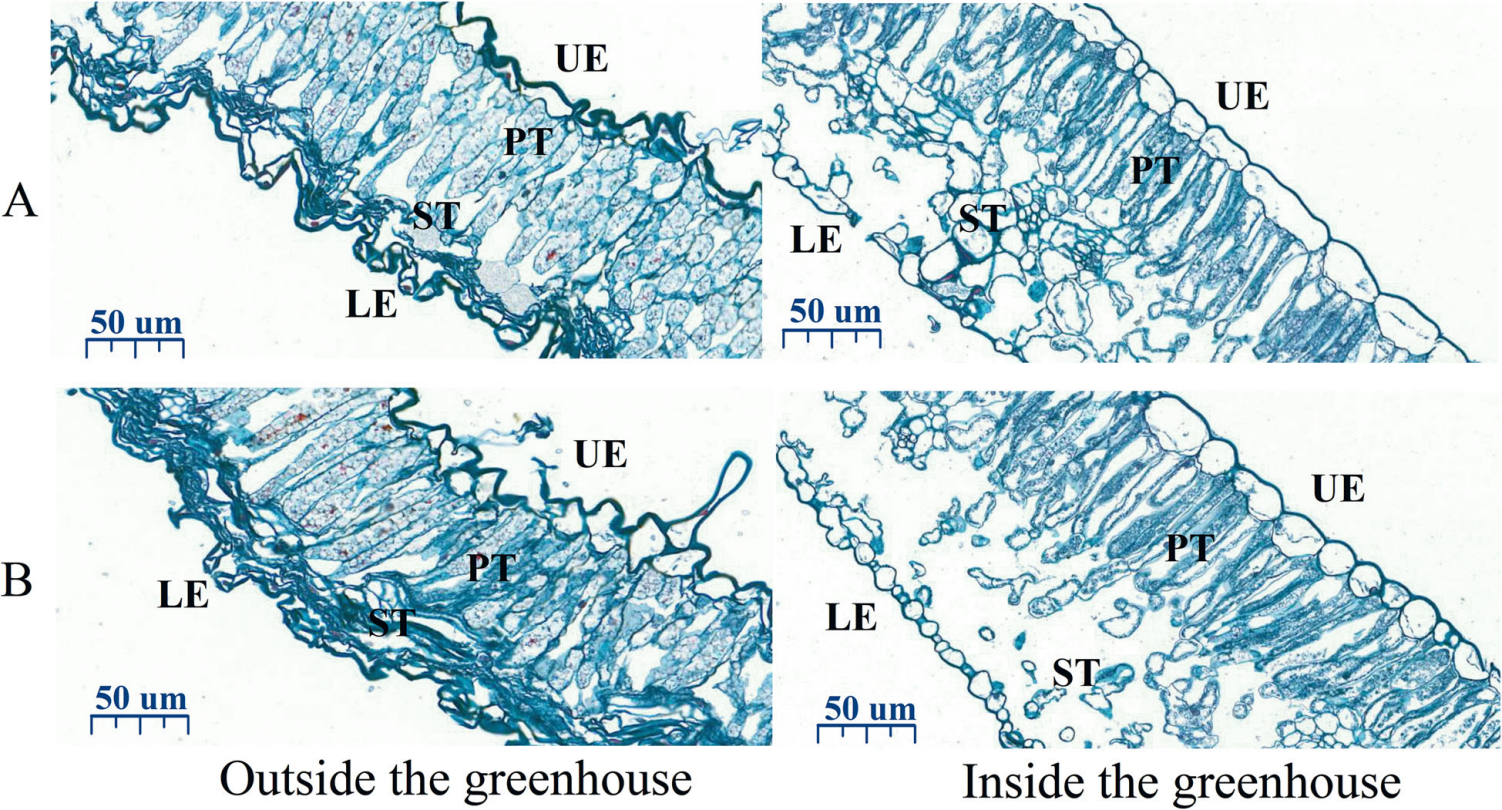
Microstructures of fresh tobacco leaves inside and outside the greenhouse. **a** is the microstructure of the first group of fresh tobacco leaves inside and outside the greenhouse. **b** is the microstructure of the second group of fresh tobacco leaves inside and outside the greenhouse. UE, upper epidermis; PT, palisade tissue; ST, sponge tissue; LE, lower epidermis

Cold stress in the field had a significant effect on the tissue structure of tobacco leaves (Fig. 4). Compared with inside the greenhouse, the upper and lower epidermis of outside the greenhouse was poor, some of the epidermis cells were separated, degenerated and disintegrated, and the sponge tissue shrunk obviously, and the leaf tissue structure cells were greatly damaged (Fig. 4). The thicknesses of laminae, upper epidermis, lower epidermis, palisade tissues, and spongy tissues of fresh tobacco leaves outside the greenhouse were significantly decreased (*P* < 0.05) (Table 1).

**Table 1:** Parameters of microstructures of fresh tobacco leaves inside and outside the greenhouse

Treatment	Thickness of lamina (μm)	Thickness of upper epidermis (μm)	Thickness of lower epidermis (μm)	Thickness of palisade tissues (μm)	Thickness of spongy tissues (μm)	Ratio of thickness of palisade tissues to that of spongy tissues (tissue ratio)
Inside the greenhouse	335.09A	30.37A	30.72A	110.31A	162.12A	0.68A
Outside the greenhouse	133.10B	15.82B	9.53B	53.76B	58.76B	0.91A

Different capital letters indicate significant differences between different treatments at the same stage (*P* < 0.05). Values represent the averages of three biological replicates

### Comparison of water loss rates of tobacco leaves inside and outside the greenhouse in the flue-curing process

**Fig. 5:**
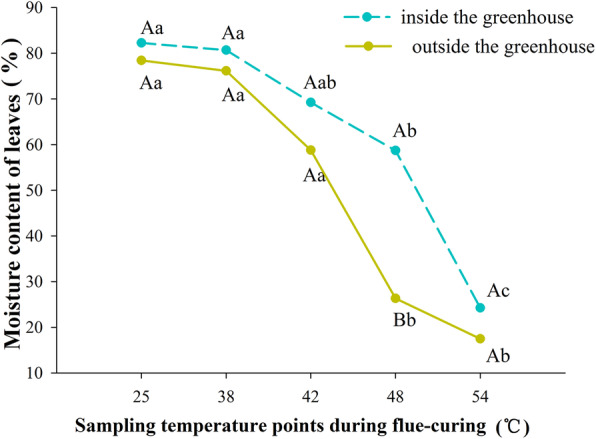
Change in the moisture content of tobacco leaves inside and outside the greenhouse at different flue-curing temperatures. Different capital letters indicate that there were significant differences among different treatments at the same sampling temperature point. Different lowercase letters indicate that there were significant differences among different sampling temperature at the same treatments (*P* < 0.05). The values were the mean of three biological replicates

**Fig. 6:**
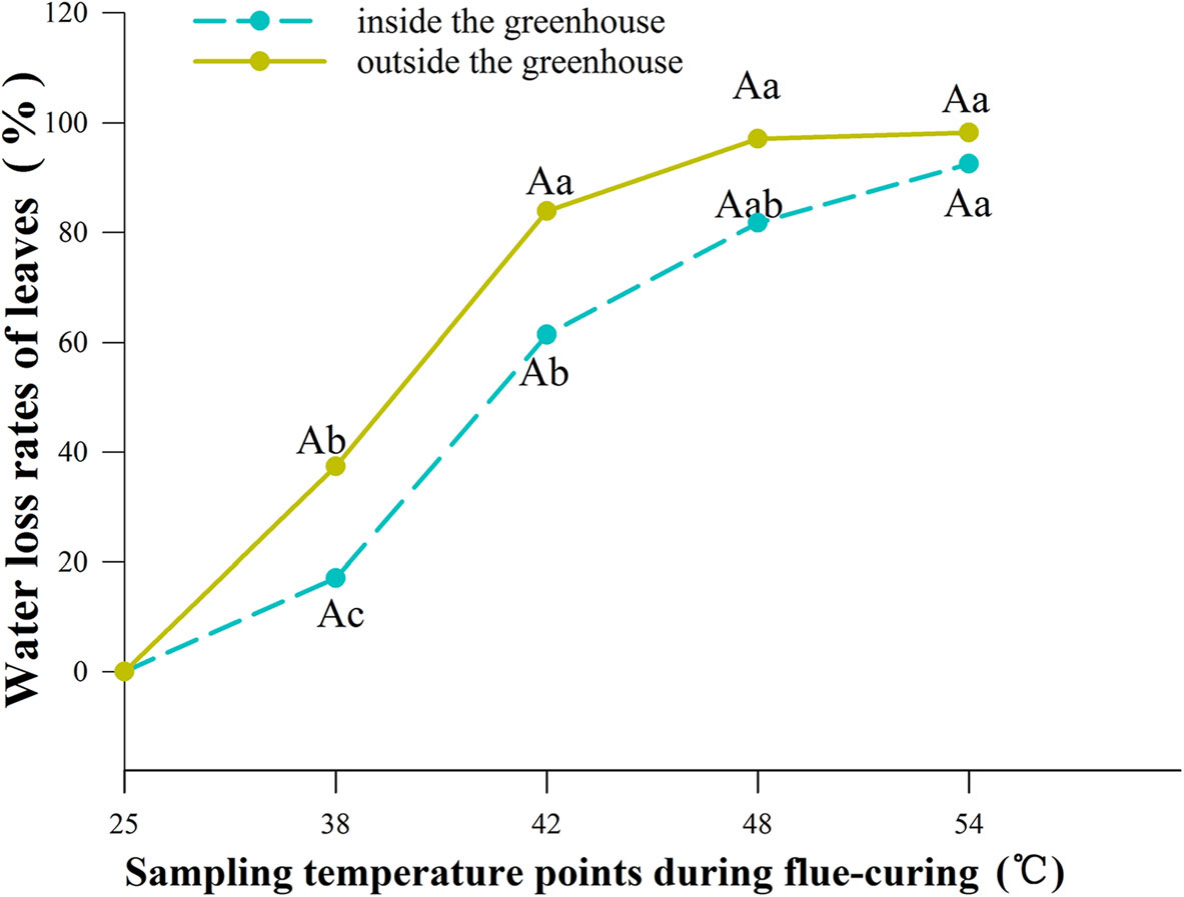
Rate of water loss from tobacco leaves inside and outside the greenhouse at different flue-curing temperatures. Different capital letters indicate that there were significant differences among different treatments at the same sampling temperature point. Different lowercase letters indicate that there were significant differences among different sampling temperature at the same treatments (*P < 0.05*). The values were the mean of three biological replicates

The moisture content of tobacco leaves inside the greenhouse was generally higher than that outside, while the water loss rate of the former was lower than that of the latter. At different curing stages, there were significant differences (*P* < 0.05) in moisture content (Fig. 5) and water loss rate (Fig. 6) of tobacco leaves. At the same curing stage, however, only at 48 °C was there a significant difference (*P* < 0.05) in moisture content of tobacco leaves inside or outside the greenhouse. There was no treatment effect on water loss rate.

### Comparison of SPAD values and Plamochromic pigments in tobacco leaves inside and outside the greenhouse during flue-curing

The SPAD values and plamochromic pigments in tobacco leaves inside and outside the greenhouse in different flue-curing stages were significantly different (*P* < 0.05) (Table 2).

**Table 2:** SPAD values and plamochromic pigments in tobacco leaves inside and outside the greenhouse at various stages of flue-curing

Treatment	Sampling temperature (°C)	Continuous curing time (h)	SPAD	Chlorophyll a (μg g^− 1^)	Chlorophyll b (μg g^− 1^)	Lutein (μg g^− 1^)	β-carotene (μg g^− 1^)
Inside the greenhouse	Fresh tobacco leaves	–	36.2Aa	286.06Aa	202.99Aa	150.30Ab	1940.38Ab
	38	23.5	14.9Ab	105.95Ab	42.72Ab	248.59Aa	3657.98Aa
	42	16.5	9.8Abc	64.20Ab	26.80Ab	297.14Aa	4127.06Aa
	48	15	8.0Abc	45.77Ab	17.01Ab	245.15Aab	4489.59Aa
	54	22.5	6.2Ac	13.33Ab	4.40Ab	206.64Aab	3524.01Aa
	Initially flue-cured tobacco leaves	–	–	43.75Ab	17.42Ab	249.00Aa	3794.68Aa
Outside the greenhouse	Fresh tobacco leaves	–	30.6Ba	114.91Ba	87.18Ba	93.28Aa	1207.53Aa
	38	23.5	13.4Ab	13.37Aa	7.94Ab	118.40Ba	1573.64Ba
	42	16.5	9.5Abc	5.12Aa	3.68Ab	112.31Ba	1669.27Ba
	48	15	7.8Abc	4.83Aa	3.50Ab	115.36Ba	1800.61Ba
	54	22.5	3.0Ac	3.88Aa	2.53Ab	139.42Aa	1888.94Ba
	Initially flue-cured tobacco leaves	–	–	6.74Aa	4.83Ab	122.23Ba	1783.31Ba

Different capital letters indicate that there were significant differences among different treatments at the same sampling temperature point. Different lowercase letters indicate that there were significant differences among different sampling temperature at the same treatments (*P < 0.05*). The values were the mean of three biological replicates

In the flue-curing process, the values of the SPAD, chlorophyll a, and chlorophyll b in tobacco leaves inside the greenhouse were all greater than those outside the greenhouse. The SPAD values of tobacco leaves inside the greenhouse were 30 to 173% greater than those outside the greenhouse. The values of the chlorophyll a in tobacco leaves inside the greenhouse were 149 to 1154% greater than those outside the greenhouse. The values of the chlorophyll b content in tobacco leaves inside the greenhouse were 74 to 628% greater than those outside the greenhouse.

The values of the lutein and β-carotene content in tobacco leaves inside the greenhouse were all greater than those outside. The two indices inside the greenhouse reached their maxima separately at 42 °C and 48 °C, respectively while outside lutein and β-carotene content each maximized at 54 °C. The content of β-carotene in tobacco leaves inside the greenhouse was 61 to 149% greater than that outside.

### Comparison of chemical composition and polyphenols in tobacco leaves inside and outside the greenhouse during curing

Chemical composition indexes and polyphenols all presented significant differences for tobacco grown inside and outside the greenhouse (Table 3).

**Table 3:** The contents of chemical substances and polyphenols in tobacco leaves inside and outside the greenhouse at different curing stages

Treatment	Sampling temperature (°C)	Continuous flue-curing time (h)	Conventional chemical compositions (%)										Polyphenols (mg g^− 1^)					
			Total sugar	Reducing sugar	Total nitrogen	Nicotine	Potassium oxide	Water soluble chloride	Starch	Protein	Sugar–nicotine ratio	Nitrogen–nicotine ratio	Neochlorogenic acid	Chlorogenic acid	Caffeic acid	Scopoletin	Rutin	Kaempferol
Inside the greenhouse	Fresh tobacco leaves	–	9.36Ac	5.20Ab	1.81Ac	2.33Aa	1.93Ab	0.12Ab	33.00Aa	8.46Aa	4.35Aa	0.82Aab	1.56Ac	7.25Bc	0.12Aab	0.12Aab	9.69Ab	0.11Ab
	38	23.5	30.07Aa	16.00Ba	2.26Aab	3.44Aa	2.75Aab	0.24Aa	4.20Bb	7.10Ab	9.23Ba	0.67Aab	3.31Aab	16.04Ba	0.19Aa	0.09Ab	12.38Aab	0.32Aa
	42	16.5	18.79Bbc	11.53Bab	2.63Aa	3.18Aa	3.57Aa	0.14Aab	1.40Ab	7.84Aab	6.04Ba	0.83Aab	2.71Ab	7.44Ac	0.05Ab	0.20Aa	8.42Ab	0.31Aa
	48	15	21.65Bab	11.98Bab	2.39Aab	2.80Aa	2.98Aa	0.18Aab	2.44Ab	7.70Aab	7.84Ba	0.85Aab	3.60Aab	12.35Aabc	0.23Aa	0.10Ab	11.52Aab	0.15Ab
	54	22.5	20.48Bab	10.18Bab	2.19Abc	3.35Aa	3.44Aa	0.14Aab	1.03Ab	7.26Aab	6.58Ba	0.66Ab	4.01Aa	14.62Aab	0.21Aa	0.16Aab	14.06Aa	0.16Ab
	Initially flue-cured tobacco leaves	–	21.32Aab	12.64Bab	2.32Aab	2.75Aa	3.12Aa	0.19Aab	1.27Ab	7.45Aab	7.63Aa	0.87Aa	3.43Aab	10.42Abc	0.23Aa	0.10Ab	9.59Bb	0.15Ab
Outside the greenhouse	Fresh tobacco leaves	–	7.60Ab	3.79Ab	1.53Ab	2.12Aa	1.43Aa	0.06Aa	39.94Aa	6.87Ba	3.61Ab	0.73Aa	1.30Ab	14.15Abc	0.21Aab	0.13Aab	11.1Aa	0.10Ab
	38	23.5	36.95Aa	25.06Aa	1.44Bb	1.89Ba	2.12Aa	0.14Aa	13.25Ab	5.41Bb	20.74Aa	0.78Aa	2.18Bab	23.20Aa	0.25Aa	0.06Ab	14.18Aa	0.19Ba
	42	16.5	36.07Aa	27.56Aa	1.71Bab	2.24Aa	1.70Ba	0.06Aa	8.72Abc	6.08Bab	16.74Aa	0.78Aa	1.29Bb	9.75Ac	0.10Ab	0.18Aa	10.62Aa	0.20Ba
	48	15	33.2Aa	23.02Aa	1.99Ba	2.86Aa	1.76Ba	0.07Ba	7.41Abc	6.56Aab	14.07Aa	0.79Aa	3.07Aa	16.73Ab	0.25Aa	0.12Aab	13.28Aa	0.08Ab
	54	22.5	36.31Aa	23.23Aa	1.64Bab	2.12Ba	1.73Ba	0.09Aa	5.40Ac	5.67Bab	17.55Aa	0.78Aa	2.82Ba	18.60Aab	0.19Aab	0.06Bb	12.92Aa	0.10Ab
	Initially flue-cured tobacco leaves	–	30.88Aa	24.42Aa	2.00Aa	2.58Aa	2.07Ba	0.06Ba	8.48Abc	6.82Aa	12.11Aab	0.79Aa	2.62Aa	15.69Ab	0.29Aa	0.11Aab	14.37Aa	0.08Ab

Different capital letters indicate that there were significant differences among different treatments at the same sampling temperature point. Different lowercase letters indicate that there were significant differences among different sampling temperature at the same treatments (*P < 0.05*). The values were the mean of three biological replicates

With increased flue-curing time, the starch contents of tobacco leaves inside and outside the greenhouse gradually decreased, exhibiting the fastest reduction in fresh tobacco leaves at 38 °C; the total sugars, reducing sugars, and sugar–nicotine ratio also reached a peak at 38 °C. Various indices used to classify tobacco leaves were greater outside the greenhouse than inside. The starch contents of tobacco leaves inside and outside the greenhouse separately varied from 1 to 33% and 5 to 40%, respectively in which the index value of tobacco leaves outside the greenhouse was 21 to 568% greater than that inside. The minimum and the maximum values separately appeared in fresh tobacco leaves and initially flue-cured tobacco leaves. The analyses of the total sugars, reducing sugars, and sugar-nicotine ratio were similar to that for starches.

The chlorogenic acid and rutin content in tobacco leaves inside and outside the greenhouse gradually increased, as described above.

### Comparison of activities of antioxidant enzymes and PPO in tobacco leaves inside and outside the greenhouse during flue-curing

**Fig. 7:**
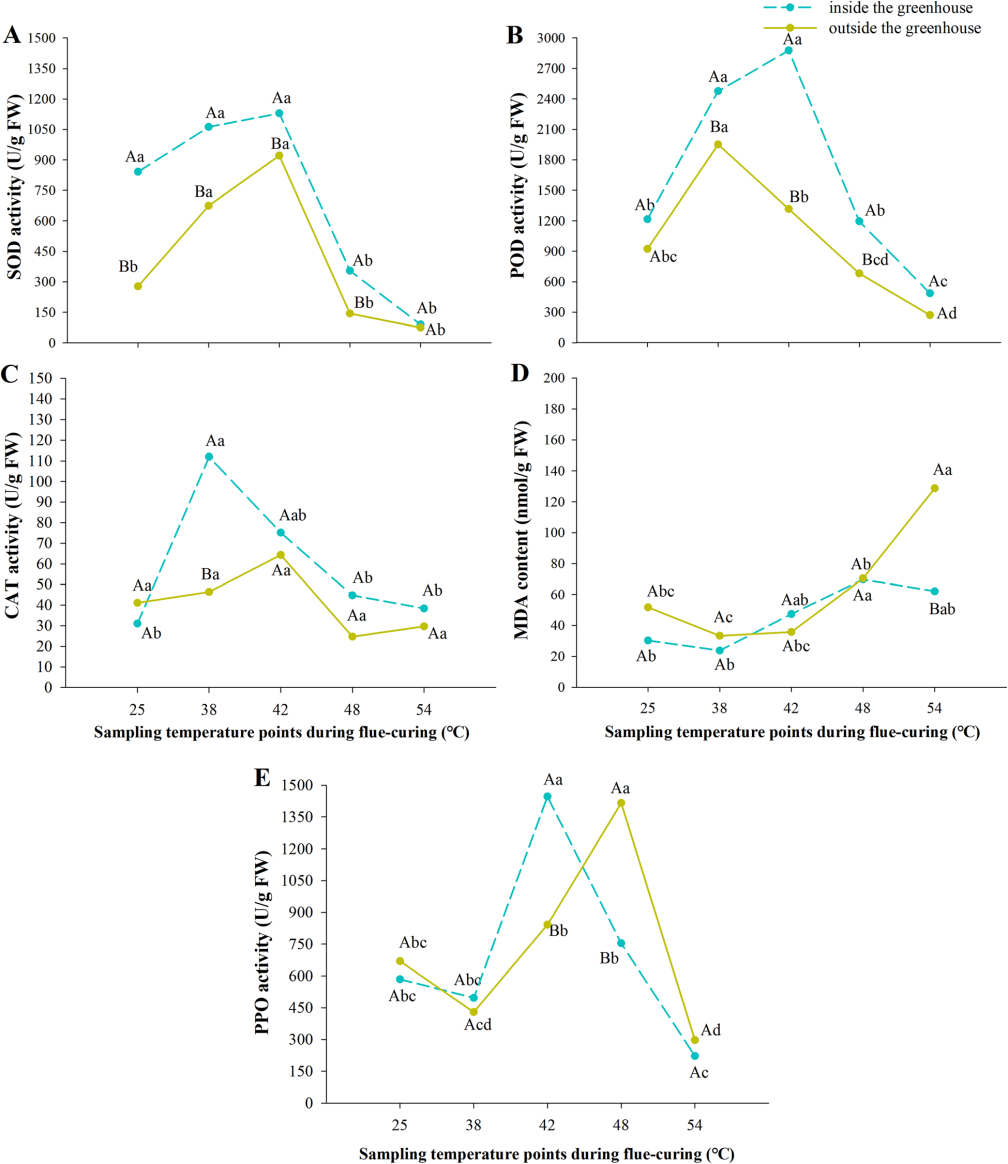
Antioxidant enzyme system and polyphenol oxidase activity inside and outside the greenhouse in flue-cured tobacco during bulk curing. Different capital letters indicate that there were significant differences among different treatments at the same sampling temperature point. Different lowercase letters indicate that there were significant differences among different sampling temperature at the same treatments (*P < 0.05*). The values were the mean of three biological replicates

The activities of SOD, POD and CAT, in the greenhouse were higher than that outside the greenhouse (Fig. 7). The activity of the three enzymes was highest at the stage of curing at 38 °C to 42 °C. In the same curing time, the SOD, POD and CAT enzymes measured in the greenhouse maintained high activity compared to those treated outside the greenhouse. The content of MDA in the treatment inside and outside the greenhouse increased with the progress of curing, and outside the greenhouse increased sharply after 42 °C and exceeded that inside the greenhouse at 48 °C. The PPO inside and outside the greenhouse treatment slightly had points of rapid increase and decrease with time of curing.

### Comparison of economic traits and sensory evaluation of initially flue-cured tobacco leaves inside and outside the greenhouse

**Fig. 8:**
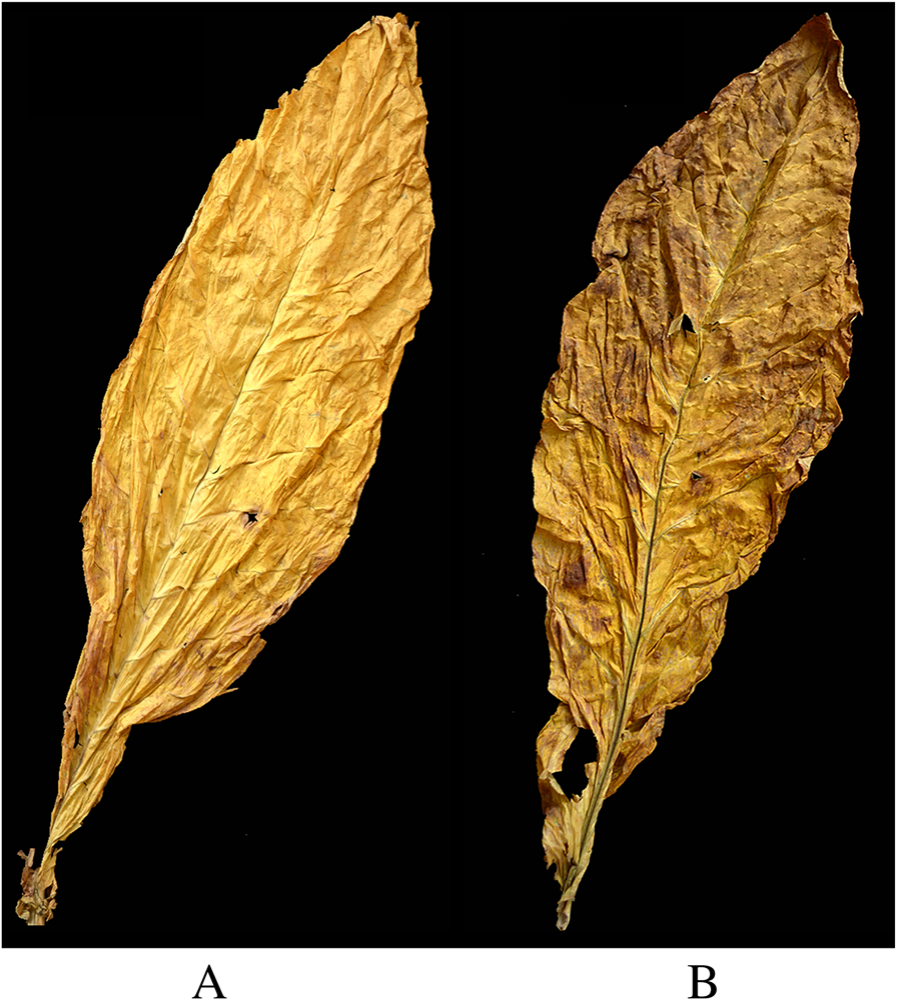
Comparison of appearance of flue-cured tobacco after curing from leaves inside and outside greenhouses. **a** Tobacco leaves of inside the greenhouse after curing. **b** Tobacco leaves of outside the greenhouse after curing

There were obvious differences in the appearance of the first-cured tobacco leaves inside and outside the greenhouse (Fig. 8). There was bright color, good opening, no obvious miscellaneous color, and browning from leaves inside the greenhouse. However, outside the greenhouse, tobacco leaves had gray and dark color, small openings, obvious miscellaneous color, and hanging ash on the surface.

There were significant differences in the yield, output value, and average price of tobacco leaves inside and outside the greenhouse (Table 4). Each index shows inside the greenhouse was higher than outside the greenhouse, and the yield inside the greenhouse was 13, 47, 37 and 30% higher than outside the greenhouse, respectively. The total score of sensory quality of tobacco leaves in the greenhouse was significantly higher than that of tobacco leaves outside the greenhouse by 20% (Table 5).

**Table 4:** Economic traits of flue-cured tobacco from inside and outside greenhouses

Treatment	Yield (kg ha^− 1^)	Output values (dollar ha^− 1^)	Proportions of middle- high-class tobacco (%)	Average price (dollar kg^−1^)
inside the greenhouse	2327A	8179A	73A	3.52A
outside the greenhouse	2051B	5552B	53A	2.71B

Different capital letters indicate significant differences under different treatments (*P* < 0.05). Values represent the averages of three biological replicates

**Table 5:** Sensory evaluation of smoking quality of flue-cured tobacco from inside and outside greenhouses

Treatment	Aroma note (10)	Aroma quality (15)	Aroma volume (15)	Concentration (10)	Mixed gas (10)	Irritancy (15)	Strength (5)	Cleanness (10)	Moisture (5)	Taste (5)	Total score (100)
inside the greenhouse	8.5	12.5	13.5	7	7.5	12	5	8.5	4	4	82.5A
outside the greenhouse	7.5	8.0	9.0	7.0	8.0	9.5	4.0	9.0	3.0	3.5	68.5B

Different capital letters indicate significant differences under different treatments (*P* < 0.05). Values represent the averages of three biological replicates

## Discussion

### Environmental factors behind cold stress in the harvest period of tobacco in Yunnan Province

Altitude, temperature, and rainfall are the major environmental factors inducing cold stress in the tobacco harvest period [16]. The tobacco was affected by low temperature in the planting and harvest periods in Yunnan Province. The killing cold stress frequently appeared in the harvest period of tobacco. The experimental site was located at a measured altitude of 2565 m, which was much higher than that of the main tobacco-growing areas in Yunnan Province. The monthly temperature in the harvest period changed significantly. The change in air temperatures in August was between 9.4 and 41.9 °C, this was extremely likely to trigger in situ cold stress. The temperature dropped by 25.4 °C at the experiment site on the day subjected to cold stress. Within the subsequent 6 days, the appearance and color of middle and upper tobacco leaves changed suddenly, turning from green to off-white [15], which was attributed to evidence from existing research that suggest cold stress can damage the photosynthetic system of plant leaves to reduce the content of chloroplast pigments [17]. Additionally, the high altitude also led to significant increase in ultraviolet irradiation, which probably contributed to damage of the photosynthetic system after cold stress [18]. Under low-temperature weather conditions, rainfall readily caused damage through freezing-injury and hail impact. A small amount of rainfall was detected, along with hail, on the day subjected to cold stress in the experiment spot, which was also one of reasons directly causing cold stress to tobacco.

### The effects of the field cold stress on the quality and curing characteristics of fresh leaves of tobacco in the harvest period

Cold stress can trigger the thickening, wilting due to water loss, and destruction of the photosynthetic system of tobacco leaves [19]. The quality of fresh tobacco leaves is considered to be the primary factor determining the curing characteristics and even the yield and quality of tobacco leaves, which is shown in various aspects such as microstructure, moisture, pigment, chemical composition, and enzyme activity [20]. The thickness of lamina, upper epidermis and lower epidermis, palisade tissue and sponge tissue of tobacco leaves (with cold damage) outside the greenhouse was significantly lower than that of fresh tobacco leaves (without cold damage) in the greenhouse. This may be due to the fact that the cold stress destroyed the tissue structure of leaves and led to the weakening of leaf assimilation ability [21]. The indicating that greenhouse treatment can not only improve the density and integrity of upper and lower epidermal cells of tobacco leaves under cold stress in the field, but also increase the length of palisade tissue and the looseness of sponge tissue so as to effectively increase the leaf thickness. The SPAD value and the content of chloroplast pigments of fresh tobacco leaves outside the greenhouse were both lower than those inside, indicating that chilling stress had a great effect on the plastid pigment of tobacco leaves, which would seriously reduce the content of aroma precursors and damage the quality of tobacco leaves. This is similar to the photosynthetic characteristics of tobacco changed in response to cold stress.

Flue-curing mainly aims to coordinate yellowing with of water loss tobacco leaves. The tobacco leaves yellow in the flue-curing process because the rate of degradation of xanthophyll (such as carotenoid) is much lower than that of chlorophyll, therefore, tobacco leaves turn from green to yellow. Water loss of tobacco leaves is very important for the formation of high-quality tobacco leaves under curing [22]. During the curing process of tobacco leaves, when the curing temperature increased between 42 ~ 48 °C, the cell membrane loses selective permeability. If the water content of tobacco leaves is too high (above 60%), it will aggravate the process of enzymatic browning of tobacco leaves and promote the formation of brown tobacco, thus reducing the quality of tobacco leaves [23, 24]. In this study, the water loss rate of tobacco leaves inside the greenhouse per unit time was lower than that outside. This may be because the cell plasma membrane of tobacco leaves outside the shed was damaged after chilling stress, and they had wilting symptoms of varying degrees, so the water loss was faster. The tobacco leaves undergoing cold-damage presented a decreasing chlorophyll content; moreover, the thickened leaves, coverage of the wax coat, and the shrunk palisade tissues and spongy tissues caused tobacco leaves to be faced with difficulties in water removal during flue-curing.

### The effects of cold stress on the physiological and biochemical characteristics of tobacco leaves in the harvest period

The change of the quality and curing characteristics of fresh tobacco leaves under cold stress was attributed to the fact that many free radicals damaged the plasma membrane system and disordered the activity of enzymes in tobacco leaves [25]. Important antioxidant enzymes in plants, SOD, POD, and CAT can eliminate excessive superoxide anions and H_2_O_2_ in plants to decrease the accumulation of the active oxygen in plants, thus reducing the accumulation of the product of membrane lipid peroxidation, that is, MDA, in plants [26]. These enzymes were closely related to stress resistance. MDA is an important product of membrane lipid peroxidation in plant cells, which reflects the integrity of plant cell membrane and the ability to resist stress [27]. When the flue-cured tobacco received the low temperature signal, the SOD activity of the tobacco leaves increased rapidly, then synergy with CAT and POD, the O_2_ and H_2_O_2_ produced by superoxides stimulated by chilling stress were decomposed subsequently [28]. With the aggravation of chilling injury, the activity of antioxidant enzymes was inhibited and could not complete the decomposition function, until the accumulated oxides in the cells poisoned the cell membrane and caused damage to the plant [29].

The results showed that the activities of POD, SOD and CAT of tobacco leaves in greenhouse were higher than those outside greenhouse, while the content and growth rate of MDA in tobacco leaves were lower than those outside greenhouse, which indicated that tobacco leaves outside greenhouse suffered obvious chilling stress, which led to the increase of cell membrane permeability and the destruction of physiological and biochemical environment. The curing process is a man-made stress environment. Under the same curing time, SOD, POD and CAT enzymes treated in the greenhouse can maintain high activity more effectively than those treated outside the greenhouse. As the main enzymes against reactive oxygen free radicals, they play an important role in alleviating cell senescence [30, 31], which can reflect that the tobacco leaves in the greenhouse have stronger stress resistance than the tobacco leaves outside the greenhouse. After cold stress, due to the damage of antioxidants system at the time of leaf harvest. After tobacco leaves enter the curing barn, under high temperature and humidity curing conditions, the cell membrane of tobacco leaves was quickly damaged, and the polyphenols in vacuoles and PPO in plastids are exposed, which could increase the synthesis of oxidized materials of phenolic compounds, thus reducing the quality of flue-cured tobacco leaves [32–34]. The peak values of tobacco leaves inside and outside the greenhouse appeared at 42 °C and 48 °C respectively, indicating that the PPO of tobacco leaves outside the greenhouse was very active. It was possibly one of the more important reasons why scalded tobacco was more likely to be found after treatment outside the greenhouse subjected to cold stress than tobacco leaves inside the greenhouse.

### The effects of the cold stress on the yield and quality of tobacco leaves in the harvest period

Conventional chemical composition, polyphenols, and neutral aroma constituents in tobacco leaves are important factors determining the yield and the quality of the flue-cured leaves [35, 36]. Cold stress affects the formation of the high-quality tobacco leaves. The content of carbon metabolites of tobacco leaves outside the greenhouse was significantly higher than that of tobacco leaves inside the greenhouse, while the content of nitrogen metabolites was the reverse, indicating that the carbon metabolism of tobacco leaves outside the greenhouse was more active and nitrogen metabolism was inhibited within cold stress. This may be due to the reason that the activation of carbon metabolism pathways is beneficial to stress resistance and alleviation [37]. Under normal circumstances, in the later growth stage of flue-cured tobacco production, cold stress promoted carbon metabolism and weakened the pathway of nitrogen metabolism, resulting in a decrease in the absorption of nitrogen and other trace elements in flue-cured tobacco. Seriously affected the process of carbon and nitrogen metabolism.

Chlorogenic acid and rutin are important polyphenols in tobacco leaves, and their contents are closely related to the aroma and taste of flue-cured tobacco leaves [38]. In this study, the contents of chlorogenic acid and rutin in tobacco leaves inside the greenhouse were much greater than those outside. And the economic characters and sensory evaluation quality of flue-cured tobacco leaves in the greenhouse were significantly higher than those outside the greenhouse (Table 4 and Table 5). This suggested that the field cold stress exerted a significant negative influence on the yield and the quality of tobacco leaves. The initially flue-cured tobacco leaves subjected to cold stress containing large areas of browned leaves from the perspective of appearance quality, thus causing the proportions of middle and high-class tobacco to be reduced on the one hand; on the other hand, owing to the internal substances not being completely transformed, a poor sensory smoking quality was found. The combination of these two features resulted in a huge loss of the yield and the quality of the initially flue-cured tobacco leaves. This showed that the economic loss of tobacco growers under cold stress in Laojun Mountain Town in 2019 was caused by the aforementioned reasons.

## Conclusions

In Yunnan tobacco-growing areas at an altitude above 2450 m, we found that flue-cured tobacco at maturity stage was vulnerable to cold stress when the daily temperature difference was greater than 20 °C and accompanied by rainfall. Through the study, we summarized the effects of field cold stress on flue-cured tobacco as follows: first, cold stress reduced the quality of fresh flue-cured tobacco leaves. Second, the tobacco leaves performed extremely poorly during the curing process. Finally, the appearance quality, chemical quality, and yield of the flue-cured tobacco leaves were severely affected. We hypothesize that cold stress caused serious damage to the plasma membrane system of flue-cured tobacco, destroyed the antioxidant enzyme system. Therefore, the quality of fresh tobacco leaves, the content of key chemical components, and the production quality were greatly reduced by cold stress. This study not only confirmed that cold stress in high-altitude tobacco areas was the main reason for the browning of tobacco leaves during the tobacco curing process. At the same time, it would provide us the basic understanding of how do fresh leave and harvest leave in flue-curing process behave differently after natural chilling stress.

## Methods

### Experimental materials

The experiment was performed in Jianji village (E 99°33′, N 26°31′, at an altitude of 2565 m) in Laojun Mountain Town in 2019. The tobacco variety HD (a conventional commercial tobacco variety) was provided by Yuxi Zhongyan Tobacco Seed Co., Ltd., China. Seedlings were produced by utilizing floated technology. Tobacco seedlings were transplanted on 13 April, with the row and plant spacing being 120 cm × 60 cm. The 15 or 16 leaves of tobacco plants were left by topped on 10 June; the tobacco leaves were manually picked and cured from the lower leaves to the upper leaves on 3 July and completed on 7 September. The loam soil chemical properties were as follows: pH was 6.5, organic matter, 56.2 g kg^− 1^; total N, 2.8 g kg^− 1^; total P 1.1 g kg^− 1^; total K, 17.6 g kg^− 1^; soluble nitrogen, 211 mg kg^− 1^; available phosphorus, 91 mg kg^− 1^; rapidly available potassium, 286 mg kg^− 1^. The fertilization situation during the experiment was as follows: the base fertilizer was compound fertilizer (150 kg ha^− 1^, N: P_2_O_5_: K_2_O = 12: 10: 25) accompanied composted farmyard manure (15,000 kg ha^− 1^). During top-dressing, compound fertilizer designed for tobacco (75 kg ha^− 1^), nitrogen fertilizer (9 kg ha^− 1^) and accompanying potassium sulfate (30 kg ha^− 1^, 51%) were applied at 15 d and 30 d after transplanting, respectively. Tillage and other agronomic practices were followed local extension recommendation.

### Experimental design

Two treatments were used after tobacco leaves were topped. Treatment 1 was inside a plastic greenhouse: a location with a length of 10 m and width of 5 m was randomly selected in the field to situate a greenhouse made of a steel frame, the top of which was covered with polyvinyl chloride (PVC) and polyethylene plastic for heat preservation, waterproof, and light transmissible. Two to three layers of shading nets distributed in the periphery of the greenhouse preserved heat and kept out wind where the guard rows were set. Treatment 2 was outside the greenhouse and was conducted in the field environment under natural conditions. A block with the same area as treatment 1 was stochastically selected, in the periphery of which guard rows were distributed. The two treatments were located in the same field block. There were at least 60 tobacco plants in each treatment. The greenhouse was installed when the cold stress comes, so the growth environment of flue-cured tobacco inside and outside the greenhouse was completely consistent before cold stress. A TH12R-EX recorder for humidity and temperature (Shenzhen Huahanwei Science and Technology Co., Ltd) was placed inside and outside the greenhouse to record changes of daily temperature and humidity. In addition, the daily meteorological data (wind speed, precipitation, temperature) were recorded at WH-2310 wireless weather station (Jiaxing Misu Electronic Co., Ltd) outside the greenhouse. It was necessary to water the tobacco plants inside the greenhouse according to the natural precipitation outside the greenhouse.

**Fig. 9:**
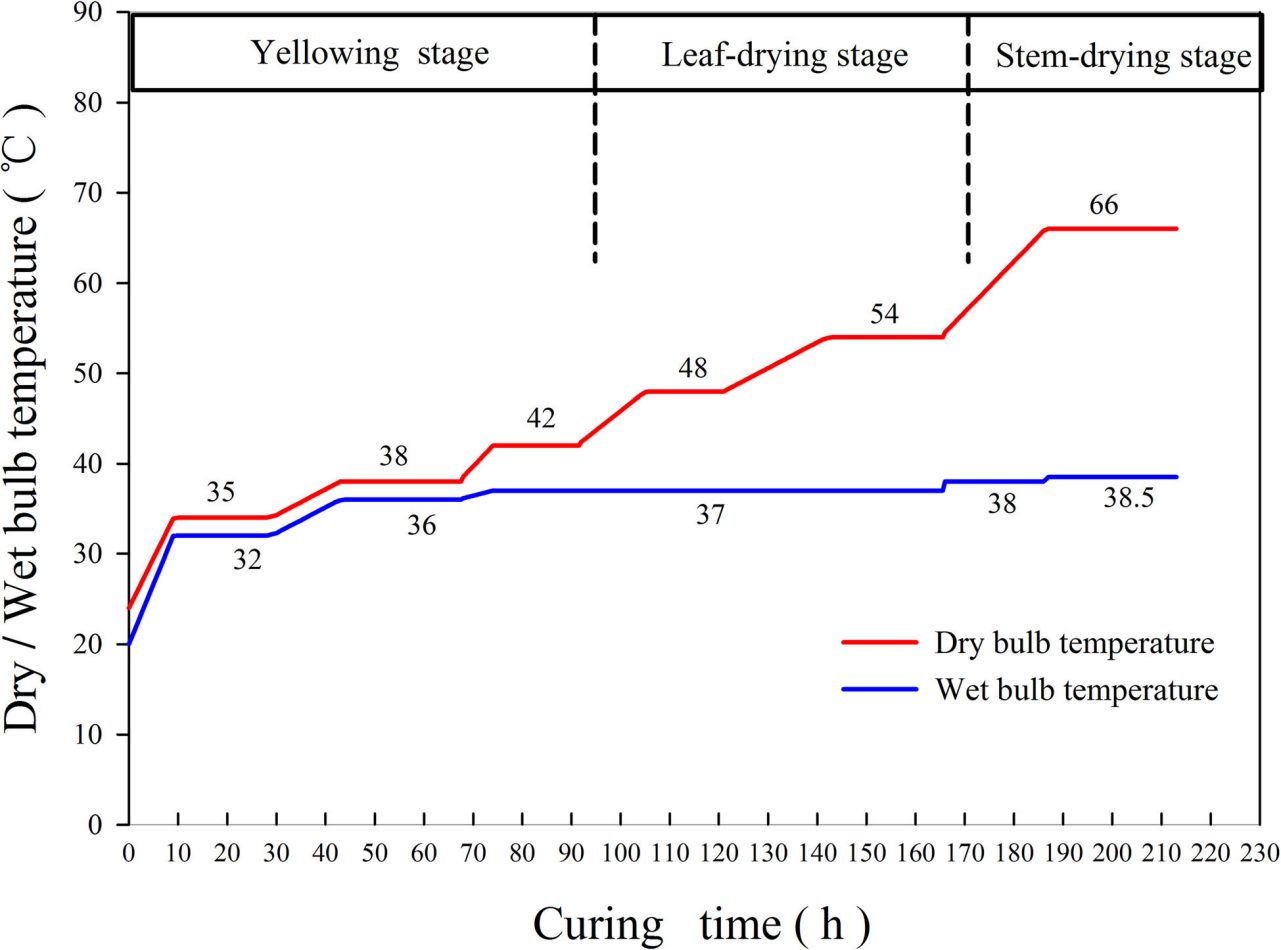
Most common curing technology for bulk curing barn in experiment tobacco-growing region

Tobacco leaves were picked and waved in the conventional harvest period for middle leaves in the local area, to ensure the equilibrium and consistency of tobacco leaf maturity and quality with moderate density. The tobacco leaves were cured by local bulk curing barn. The flue-curing was performed by the most common curing mode in the region (Fig. 9). Additionally, 100 to 120 tobacco leaves were weaved in each rod and a total of three layers were set, in each layer there were 150 to170 rods.

### Data collection and index measurement

#### Meteorological data and tobacco leaves sample acquisition

Through the weather station, the recorder for temperature and humidity, and the local meteorological bureau, the daily meteorological data (including temperature, rainfall, and wind speed) in July and August were comprehensively collected. And focus on the analysis of meteorological data during the occurrence of cold stress of tobacco leaves in the local field. Only middle leaves (5th to 10th leaves from bottom to top) of tobacco samples were picked. The samples were separately collected before the picking and curing period (fresh tobacco leaves), in critical periods (at the end of 38 °C, 42 °C, 48 °C, and 54 °C) during the flue-curing and from initially flue-cured tobacco leaves. In each stage, the various indices were determined through measure 10 pieces of tobacco leaves.

#### Microstructures of fresh tobacco leaves

A small piece of about 0.5 cm × 0.5 cm was cut between sixth and seventh branch veins from the right leaf tip to the leaf base of the fresh tobacco sample. The leaf sample was fixed with FAA (Formaldehyde, 50% Alcohol and Acetic acid) stationary liquid to prepare the slices by applying conventional paraffin methods, with the slice thickness of 10 μm; afterwards, the slices were stained by hematoxylin and sealed by Canada balsam to prepare permanent microscope mounts, which was observed and measured with an Olympus microscope. Two slices were observed over five separate views to calculate their means. The indices (including thicknesses of lamina, lower epidermis, upper epidermis, spongy tissues and palisade tissues) were measured for analysis.

#### Soil plant analysis development (SPAD) value of tobacco leaves

The SPAD values of tobacco leaves in various sampling stages were measured by a SPAD-502 chlorophyll meter (Konica Minolta, Japan) with measurement accuracy within ±1.0 SPAD unit. Three positions were separately and symmetrically measured in the left and right halves of the leaves, at the middle between the leaf margins symmetrical about the main vein and the main veins. The mean of readings represented the reading of the chlorophyll value.

#### Measurement of water loss rate and moisture content of tobacco leaves

The electronic balance (U.S. Shuangjie SA-200Y, resolution d of 0.1 g) was used to determine the weight of fresh tobacco leaves (surface is dry without moisture) and samples in various stages during curing. The determine and calculation methods of the moisture content and water loss rate of tobacco leaves in various stages during curing (including fresh tobacco leaves) refer to [15].

#### Measurement of antioxidant enzymes indices of tobacco leaves

The samples of tobacco leaves were put in liquid nitrogen immediately after being sampled at various stages and then transferred to a refrigerator at − 80 °C to measure the antioxidant enzymes therein, mainly including: SOD, POD, CAT, MDA, and polyphenol oxidase (PPO). The activities of various enzymes were measured by applying kits produced by Keming Biotechnology Co., Ltd., Suzhou, China.

#### Measurement of chemical indices of tobacco leaves

The *Tobacco and Tobacco Products–Determination of Water Soluble Sugars*–*Continuous Flow Method* (YC/T159–2002) was strictly used to measure reducing sugar and total sugar; the *Tobacco and Tobacco Products–Determination of Total Nitrogen – Continuous Flow Method* (YC/T161–2002) was used to measure total nitrogen; the *Tobacco and Tobacco Products–Determination of Nicotine–Continuous Flow Method* (YC/T160–2002) was used to measure nicotine; the *Tobacco and Tobacco Products–Determination of Starch–Continuous Flow Method* (YC/T 216–2014) was used to measure starch content. The YC/T 202–2006 was referred to measure the content of total phenols, scopoletin, the chlorogenic acid, β-carotene, neochlorogenic acid, lutein, rutin, caffeic acid.

#### Measurement of chloroplast pigment of tobacco leaves

The high-performance liquid chromatography method (YC/T382–2010) was used to determine carotenoid, chlorophyll a and chlorophyll b of tobacco sample.

#### Measurement of economic traits of initially flue-cured tobacco leaves

The International Standard GB2635–92 was used to grading the initially flue-cured tobacco leaves, and the average price was the local price of the that year. The average prices and proportions of middle- high-class tobacco were computed in different treatments.

#### Measurement of sensory quality of initially flue-cured tobacco leaves

According to the China Tobacco Yunnan Industrial Co. Ltd. making smoking standard of sensory quality for single tobacco leaves, seven experts from the China Tobacco Technology Centre in Yunnan Province was assessed the sensory quality of flue-cured tobacco leaves. The smoking standard were included: Aroma note (10), Aroma quality (15), Aroma volume (15), Concentration (10), Mixed gas (10), Irritancy (15), Strength (5), Cleanness (10), Moisture (5), Taste (5), the total score was 100.

### Data statistics

The general linear model (GLM) procedure of SAS 9.3 computer package (SAS Institute Inc., Cary, NC) was used to analyze the date. There was significance different between treatments when the *P* < 0.05. The separation of means at the 95% confidence interval was carried out using Tukey’s honest significant difference (HSD) test. The Origin 6.0 (Microcal Software, Inc. USA) and Sigma Plot 12.5 (Systat Software Inc. USA) were applied to prepare all charts.

## Supplementary Information

**Additional file 1: Fig. S1.** The variation of maximum temperature and minimum temperature in Jianchuan from April to September in 2014 to 2018.

**Additional file 2.** Contains original data of figures and tables in the manuscript.

## Data Availability

The datasets used and/or analysed during the current study are included in this published article (and its supplementary information files, Fig. S1).

## Abbreviations

PAL: Phenylalnine ammonia lyase
MDA: Malondialdehyde
POD: Peroxidase
SOD: Superoxide dismutase
CAT: Catalase
HD: Flue-cured tobacco (cultivated variety: Honghuadajinyuan)
PPO: Polyphenol oxidase
SPAD: Soil and Plant Analysis Development
